# Flexible Metal–Organic Frameworks for Adsorptive Separation of Liquid Hydrocarbons

**DOI:** 10.1021/acs.accounts.5c00217

**Published:** 2025-06-09

**Authors:** Feng Xie, Hao Wang, Jing Li

**Affiliations:** † Department of Chemistry and Chemical Biology, 242612 Rutgers University, 123 Bevier Road, Piscataway, New Jersey 08854, United States; ‡ Hoffmann Institute of Advanced Materials, 47891 Shenzhen Polytechnic University, 7098 Liuxian Boulevard, Shenzhen 518055, P. R. China

## Abstract

The separation and purification of liquid hydrocarbons are vital for producing various petrochemical feedstocks. However, the structural and chemical similarities among these hydrocarbons make conventional separation methods such as distillation and extraction both challenging and energy intensive. Adsorptive separation using porous solid adsorbents presents a promising and energy-efficient alternative. Among these, flexible metal–organic frameworks (FMOFs) as a subgroup of MOFs have emerged as an unparalleled class of porous materials for highly selective adsorption-based separation and purification of liquid hydrocarbons. Having intrinsically dynamic structures, FMOFs exhibit very different adsorption behaviors compared to rigid MOFs (RMOFs), those that do not involve structure transformations upon activation, and molecular adsorption.

In recent years, we have focused on developing high-performance FMOFs with different dimensionalities, spanning from one-dimensional (1D) chains to two-dimensional (2D) layers and to three-dimensional (3D) networks. Their structural flexibility arises from either local rotation and vibration of organic linkers or global structural changes, enabling stimuli-responsive molecular adsorption. By leveraging their distinct temperature- and adsorbate-dependent adsorption behaviors, we have achieved highly efficient separation of numerous important liquid hydrocarbons through molecular sieving, the mechanism that offers the highest selectivity. The unique dynamic structures and adsorption properties allow FMOFs to have high adsorption capacity, exceptional selectivity, and fast kinetics simultaneously, overcoming the trade-offs typically encountered by conventional adsorbents including RMOFs.

In this *Account*, we summarize our recent advances using FMOFs for the adsorptive separations of three key groups of liquid hydrocarbons: C6 alkane isomers, C8 alkylaromatic isomers, and C6 cyclic hydrocarbons. We highlight the interplay between FMOF structures and sorbate properties, focusing on their unique temperature- and adsorbate-dependent adsorption behaviors and molecular sieving mechanism responsible for high selectivity. We also discuss their inverse size-selective adsorption phenomena stemmed from adsorbate-induced structural transformations—a phenomena not possible in conventional RMOFs, which typically exhibit size-exclusion selectivity. Representative FMOFs with superior separation performance are discussed, along with their underlying working principles. Finally, we address the existing challenges and propose potential strategies to enhance their performance aiming for applications in petrochemical industry. Overall, our studies not only unveil a new dimension of flexible porous crystals but also provide a strategic framework for their design and implementation in highly selective, sieving-based molecular separation. By deepening the understanding of structure–property relationships, our findings offer valuable insights that can inspire future advancements in adsorptive separation technologies, with significant implications for the petrochemical industry and beyond.

## Graphical abstract

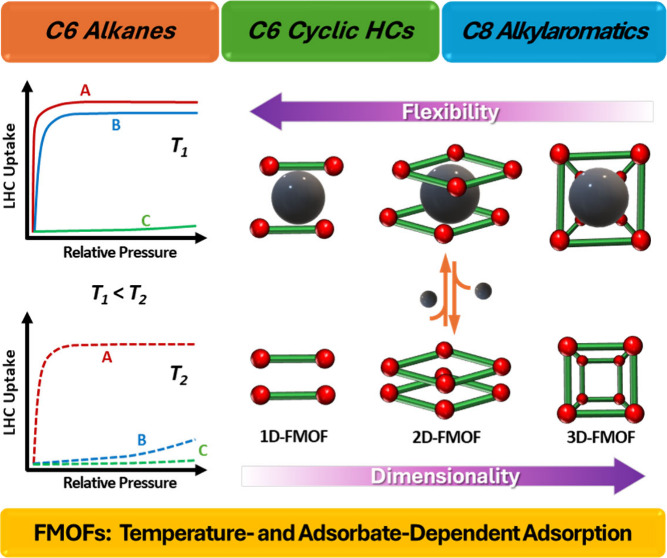

## Key References

-

Wang, H. ; Dong, X. ; Velasco, E. ; Olson, D. ; Han, Y. ; Li, J. One-of-a-kind: a microporous metal–organic framework capable of adsorptive separation of linear, mono- and dibranched alkane isomers via temperature- and adsorbate-dependent molecular sieving. Energy Environ. Sci. 2018, 11, 1226–1231 .^1^ We reported the first study on the separation of linear, monobranched, and dibranched hexane isomers into pure components by a 3D-FMOF via temperature- and adsorbate-dependent size-exclusion mechanism.
-

Li, L. ; Guo, L. ; Olson, D. ; Xian, S. ; Zhang, Z. ; Yang, Q. ; Wu, K. ; Yang, Y. ; Bao, Z. ; Ren, Q. ; Li, J. Discrimination of xylene isomers in a stacked coordination polymer. Science 2022, 377, 335–339 35857587 10.1126/science.abj7659.^2^ We demonstrated, for the first time, that a stacked 1D flexible coordination polymer can act as an ideal sorbent for molecular recognition and sieving of xylene isomers at different temperatures.
-

Lin, Y. ; Yu, L. ; Ullah, S. ; Li, X. ; Wang, H. ; Xia, Q. ; Thonhauser, T. ; Li, J. Temperature-programmed separation of hexane isomers by a porous calcium chloranilate metal–organic framework. Angew. Chem. Int. Ed. 2022, 61­(50), e202214060 10.1002/anie.20221406036261325.^3^ We developed a novel 3D-FMOF that exhibits temperature- and adsorbate-dependent adsorption of hexane isomers with different branching, enabling temperature-programmed separation of these isomers via molecular sieving.
-

Xie, F. ; Chen, L. ; Morales, E. ; Ullah, S. ; Fu, Y. ; Thonhauser, T. ; Tan, K. ; Bao, Z. ; Li, J. Complete separation of benzene-cyclohexene-cyclohexane mixtures via temperature-dependent molecular sieving by a flexible chain-like coordination polymer. Nat. Commun. 2024, 15­(1), 2240 38472202 10.1038/s41467-024-46556-6PMC10933443.^4^ We used a chain-like 1D-FMOF as an efficient adsorbent to discriminate ternary mixtures of C6 cyclic hydrocarbons via a temperature-dependent sieving mechanism.

## 1 Introduction

The petrochemical industry is a cornerstone of the global economy, converting crude oil into a vast array of products.^5^ Central to this process is petroleum hydrocarbon separations, which isolate and purify specific hydrocarbons or groups with similar properties.^6^ These separations are crucial to maximize feedstock potential and ensure efficient, sustainable petrochemical production. Particularly, purification of alkenes, separation of alkane isomers and benzene derivatives are among the seven chemical separations set to change the world.^7^ Liquid hydrocarbons (LHCs, e.g., C6–C8 species) are sorted based on their reactivity or combustibility. However, their longer-chain length, high viscosity, low vapor pressure and formation of azeotropes complicate the separation processes.^8^

Extractive distillation and adsorption are widely applied in industrial LHC separation.^9,10^ While effective, distillation is energy-intensive, leading to a high cost and greenhouse gas emissions. Extractive distillation also introduces third agents, causing impurities. Adsorptive separation, on the other hand, uses solid adsorbents to selectively capture LHCs.^10^ The efficient adsorptive separation of LHCs requires rigorous specifications regarding the pore structure and chemistry of the adsorbents. Porous materials such as zeolites, carbons, and alumina have been extensively studied for such processes.^11^ Zeolites, in particular, are benchmark adsorbent materials.^12^ For example, current industrial separation uses zeolite 5A to separate linear hexane from branched isomers via size exclusion.^13^ However, with an inherently small porosity, its adsorption capacity is relatively low, and it cannot discriminate monobranched from dibranched alkanes—crucial for achieving high research octane numbers (RONs) in premium gasoline.^14^ Given the limitations of traditional adsorbents, there is a pressing need to develop porous materials that can significantly enhance performance in separating industrially important LHCs.

Metal–organic frameworks (MOFs) have emerged as a promising class of crystalline porous materials for the separation of LHCs.^15–18^ MOFs offer distinct advantages over conventional adsorbents, including exceptionally high surface areas, systematically tunable crystal structures, and easily functionalizable pore surfaces.^19^ By applying crystal engineering principles, MOFs can be designed with precisely controlled pore structures optimal for separating specific hydrocarbons.^20^ Previous studies on LHC separations have been largely on RMOFs.^16,21,22^ Only recently have FMOFs begun to receive increased attention. Unlike RMOFs, which do not undergo structural phase transitions upon activation or molecular adsorption, FMOFs exhibit intrinsic structural flexibility, with changes that are typically triggered by some forms of external stimuli, such as light, electric field, temperature, pressure, and guest molecules.^23–25^ These structure transformations—manifesting as breathing, expanding, swelling, sliding, or gate-opening—are often reversible.^25–27^ This flexibility imparts unique adsorption behaviors of FMOFs, enabling them to outperform RMOFs with significantly enhanced separation and purification of LHCs.^17,27^ RMOFs often face a trade-off among three key performance parameters: adsorption capacity, selectivity, and diffusion rate, particularly for LHCs with very similar properties. Larger pore size and higher porosity (i.e., surface area and pore volume) typically result in high adsorption capacity but relatively low selectivity, while smaller pores and porosities improve selectivity at the cost of capacity. When the pore apertures/windows are small (e.g., in cage-like structures) but porosity remains relatively large, both high capacity and selectivity can be achieved, but often at the expense of diffusion rates. In contrast, FMOFs can eliminate this trade-off by leveraging their temperature- and adsorbate-dependent adsorption behaviors. This unique adaptability enables simultaneous enhancement of all three parameters–large capacity, high selectivity, and fast diffusion rate–through molecular sieving,^28^ positioning FMOFs as a potentially transformative solution for efficient and selective hydrocarbon separations.

This ***Account*** thus summarizes our recent research on the adsorptive separation of three groups of LHCs using FMOF adsorbents (Scheme 1): (i) C6 alkane isomers, (ii) C8 alkylaromatic isomers, and (iii) C6 cyclic hydrocarbons, each characterized by members with very similar chemical and physical properties (Table 1).^17,22,29^ The FMOFs selected as examples in the discussion vary in dimensionality, from 3D networks with lower flexibility (e.g., breathing and sliding) to lower-dimensional structures (2D and 1D) with higher flexibility (e.g., interlayer expanding and interchain swelling), which demonstrate distinct stimuli-responsive (temperature- and adsorbate-dependent) molecular adsorption behaviors arising from structural flexibility and emphasize the underlying structure–property relationships. Our discussion highlights the molecular-sieving governed separation mechanism specifically desirable for enhanced separation efficiency and provides insights into the chemistry of host–guest interactions in these systems.

**Scheme 1:**
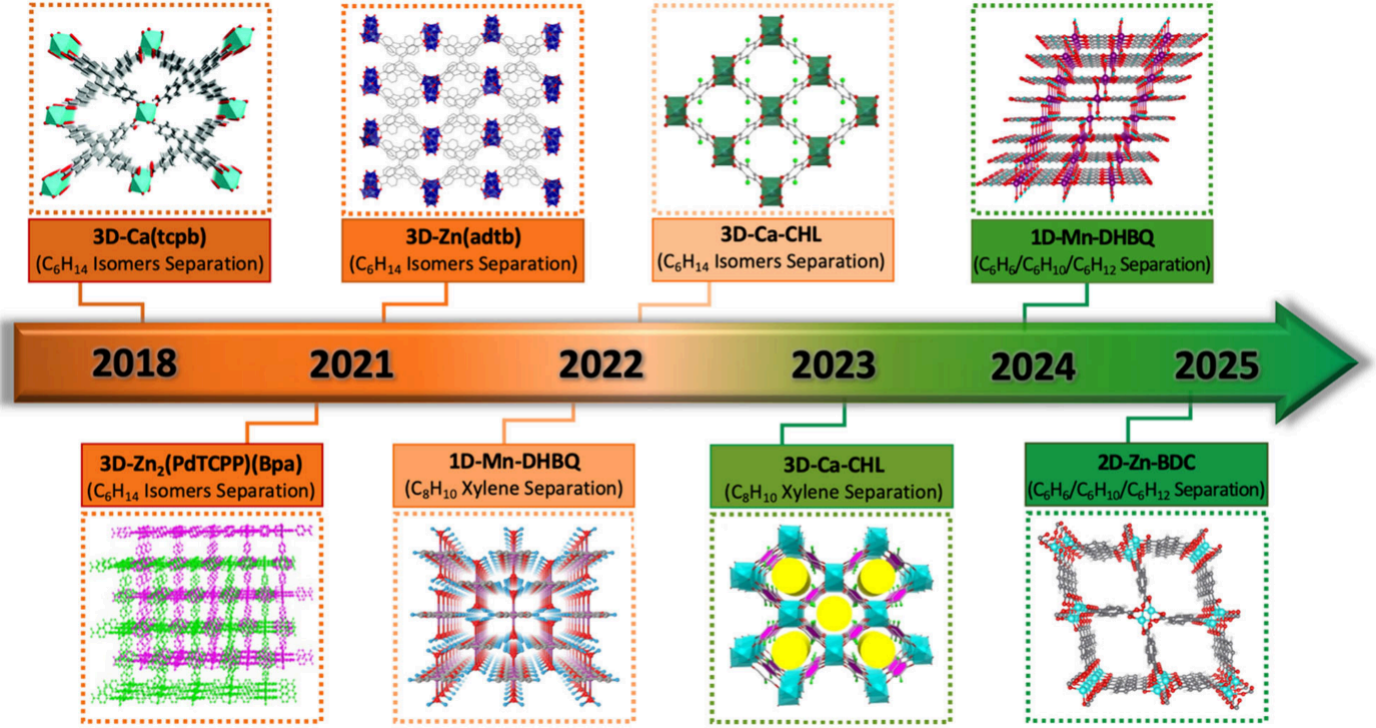
Timeline of Our Recent Progress and Important Development in LHCs Separations by FMOFs Featuring Different Dimensionalities and Various Structure Changes. Reproduced with Permission from Ref 1 (Copyright 2018, Royal Society of Chemistry), Ref 30 (Copyright 2021, Wiley-VCH), Ref 31 (Copyright 2021, American Chemical Society), Ref 2 (Copyright 2022, American Association for the Advancement of Science), Ref 3 (Copyright 2022, Wiley-VCH), Ref 32 (Copyright 2023, Wiley-VCH), Ref 4 (Copyright 2024, Springer Nature), Ref 33 (Copyright 2025, American Chemical Society)

**Table 1:** Physicochemical Properties of Liquid Hydrocarbons^18,34^

**Liquid Hydrocarbons**	**Chemical Formula**	**Boiling Point** (K)	**Molecular Dimensions** (*x*/*y*/*z*, Å^3^)	**Kinetic Diameter** (Å)
*n*-Hexane (nHEX)	C_6_H_14_	341.9	10.3 × 4.5 × 4.0	4.5
2-Methylpentane (2MP)	C_6_H_14_	334.0	9.2 × 6.4 × 5.3	5.0–5.5
3-Methylpentane (3MP)	C_6_H_14_	336.4	9.3 × 6.2 × 5.2	5.0–5.5
2,2-Dimethylbutane (22DMB)	C_6_H_14_	322.9	8.0 × 6.7 × 5.9	6.2
2,3-Dimethylbutane (23DMB)	C_6_H_14_	331.2	7.8 × 6.7 × 5.3	5.6
Benzene	C_6_H_6_	353.3	6.6 × 7.3 × 3.3	5.85
Cyclohexene	C_6_H_10_	356.1	7.0 × 6.6 × 5.0	5.85
Cyclohexane	C_6_H_12_	353.9	7.2 × 6.6 × 5.0	6.0
p-Xylene (pX)	C_8_H_10_	411.5	6.6 × 3.8 × 9.1	6.7
m-Xylene (mX)	C_8_H_10_	417.6	9.0 × 4.0 × 7.3	7.1
o-Xylene (oX)	C_8_H_10_	412.3	7.3 × 3.8 × 7.8	7.4
Ethylbenzene (EB)	C_8_H_10_	409.4	6.6 × 5.3 × 9.4	6.7

## 2 Separation of C6 Alkane Isomers

The separation of C6 alkane isomers is a critically important process in the petrochemical industry. Following catalytic isomerization, lower branched (e.g., linear and monobranched) isomers are used as feedstocks for the production of olefins. On the other hand, higher branched (e.g., dibranched) isomers with higher RONs, are the main components in premium gasoline.^16,17^ To meet the specific standards for these end uses, efficient separation of the C6 alkane isomers is essential. However, their remarkably similar physicochemical properties present significant challenges for energy consumption. Currently, zeolites such as 5A and ZSM-5 are used as benchmark adsorbents for alkane separation, but their performance remains insufficient due to relatively low adsorption capacity and selectivity.

As a subclass of MOFs, FMOFs often undergo reversible structural changes in response to external stimuli such as guest molecules and temperature, giving rise to very interesting adsorption properties different from RMOFs. Temperature-dependent adsorption behavior induced by large hydrocarbons is an interesting phenomenon. It was not explored in early studies using FMOFs for hexane isomer separation^35^ but only discovered recently.^1–4^ Within a specific temperature range, molecules with suitable size and shape and sufficiently strong interaction with the adsorbent can expand the flexible pore space, facilitating their adsorption. Conversely, molecules with somewhat different geometry, often (but not always) larger or having weaker interactions with the adsorbent are excluded across all or part of the experimental temperature range. This behavior enables separation of LHC mixtures such as C6 alkane isomers by a highly efficient molecular sieving mechanism.

In 2018, we reported the first example of temperature-dependent separation of C6 alkane isomers using a highly flexible 3D FMOF, Ca­(tcpb) (tcpb = 1,2,4,5-tetrakis­(4-carboxyphenyl)-benzene).^1^ The framework features two similar 1D open channels with pore diameters of ∼5.5 Å (Figure 1a), comparable to the kinetic diameters (KD) of branched alkanes. The estimated BET surface area is ∼220 m^2^/g, significantly lower than that of zeolite 5A (∼600 m^2^/g). Ca­(tcpb) exhibits a distinct temperature- and sorbate-dependent adsorption behavior toward C6 alkane isomers. At 120 °C, only nHEX is adsorbed, while at 60 °C, both nHEX and 3MP can be adsorbed, with 22DMB excluded at both temperatures (Figure 1b-1c). This enables a two-step molecular-sieving process: Branched isomers are separated from nHEX at 120 °C, followed by separating dibranched and monobranched isomers at 60 °C (Figure 1d). Breakthrough experiments confirmed Ca­(tcpb)’s effectiveness in separating an equimolar ternary mixture of nHEX/3MP/22DMB into pure components using a temperature-programmed two-column system (Figure 1e). The structural flexibility and adsorbate-dependent pore enlargement were examined by a detailed structure analysis of the as-made, activated, and hydrocarbon-loaded samples (Figure 1f). A clear correlation was established between the size (or degree of branching) of the C6 isomers and the extent of pore enlargement (Figure 1g-1h). The observed structural change from activated to alkane-loaded Ca­(tcpb) (primarily the elongation in the *b*-axis of the unit cell) is directly proportional to the increase of the pore diameter of Ca­(tcpb) upon adsorption of the isomers (Figure 1i). Achieving simultaneously high uptake capacity, ideal selectivity and fast adsorption kinetics, Ca­(tcpb) stands out as a promising candidate for complete separation of hexane isomers as well as similar liquid alkanes of different branches (e.g., C5 and C7 isomers).

**Figure 1:**
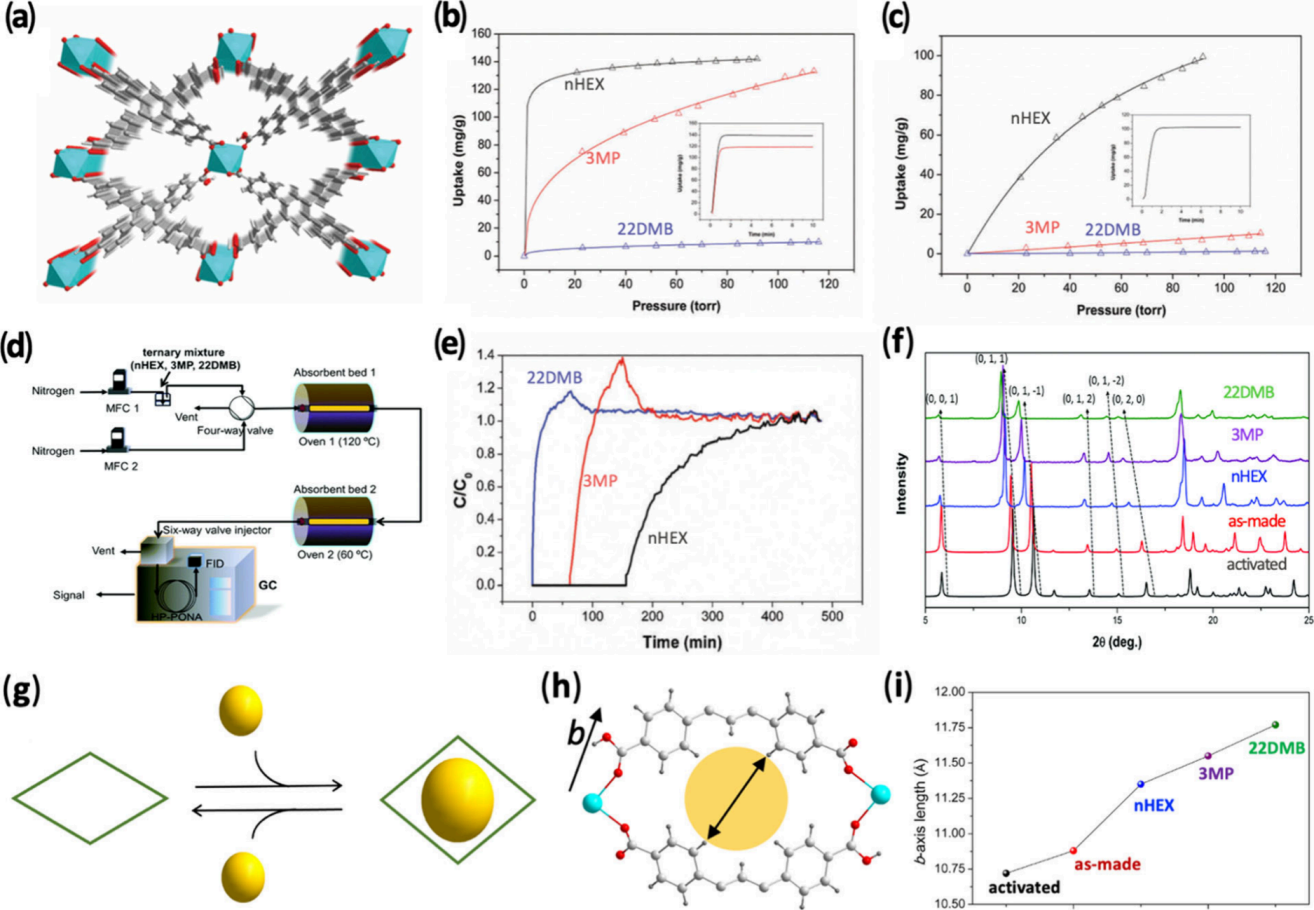
(a) Crystal structure of Ca­(tcpb). (b-c) Adsorption isotherms of nHEX, 3MP, and 22DMB at 60 and 120 °C. (d) Schematic representation and device setup of temperature-programmed separation process. (e) Breakthrough curves of Ca­(tcpb) for equimolar ternary mixture of nHEX/3MP/22DMB using a two-column setup at 120 and 60 °C. (f) PXRD patterns of different forms of Ca­(tcpb). (g) Schematic representation of the pore enlargement of Ca­(tcpb) upon guest inclusion. (h) Structure drawing showing the direct correlation between the changes in the *b*-axis and pore diameter. (i) Plotted length of the *b*-axis of various forms of Ca­(tcpb). Reproduced with permission from ref 1. Copyright 2018, Royal Society of Chemistry.

Our subsequential efforts in developing FMOF adsorbents capable of molecular-sieving based separation of C6 alkane isomers resulted in several new structures, such as Zn_2_(PdTCPP)­(Bpa) (TCPP = 5,10,15,20-tetrakis­(pentafluorophenyl)­porphyrin, Bpa = 9,10-bis­(4-pyridyl)-anthracene),^30^ Zn­(adtb) (adtb = 4,4′,4″,4‴-(anthracene-9,10-diylidenebis­(methanediylylidene)) tetrabenzoic acid),^31^ and Ca-CHL (HIAM-203, CHL = chloranilic acid),^3^ all of which are 3D networks. Similar to Ca­(tcpb), Ca-CHL exhibits temperature-dependent adsorption behavior, enabling the separation of hexane isomers via molecular sieving. This structure features 1D channels of larger pore size (∼6 Å) decorated with chloro groups and a higher BET surface area (499 m^2^/g) than Ca­(tcpb) (Figure 2a). While the crystal structure determination of the activated and hexane isomer loaded Ca-CHL was unsuccessful, we speculate its pore opening process assembles that of Ca­(tcpb) based on the PXRD analysis of the samples in different forms.^1^ Its structural flexibility allows for selective accommodation of C6 alkanes with different degrees of branching. At 30 °C, Ca-CHL adsorbs a significant amount of linear nHEX and monobranched 3MP while excluding dibranched 22DMB (Figure 2b). At 150 °C, it further excludes 3MP, selectively adsorbing only nHEX (Figure 2c). Interesting to note is that upon adsorption, the C6-loaded structures convert back to that of the as-synthesized form, as evident from the PXRD analysis (Figure 2d). This is different from Ca­(tcpb), for which the structures of C6-loaded samples vary notably from that of the as-synthesized sample and correlate to the size of isomers. Temperature-programmed two-column breakthrough experiments (set at 150 and 30 °C in sequence) confirmed the complete splitting of the individual isomers (Figure 2e). This study not only underscores the potential of Ca-CHL for cost-effective separation of LHCs but, more importantly, validates the temperature-responsive adsorption behavior induced by structural flexibility.

**Figure 2:**
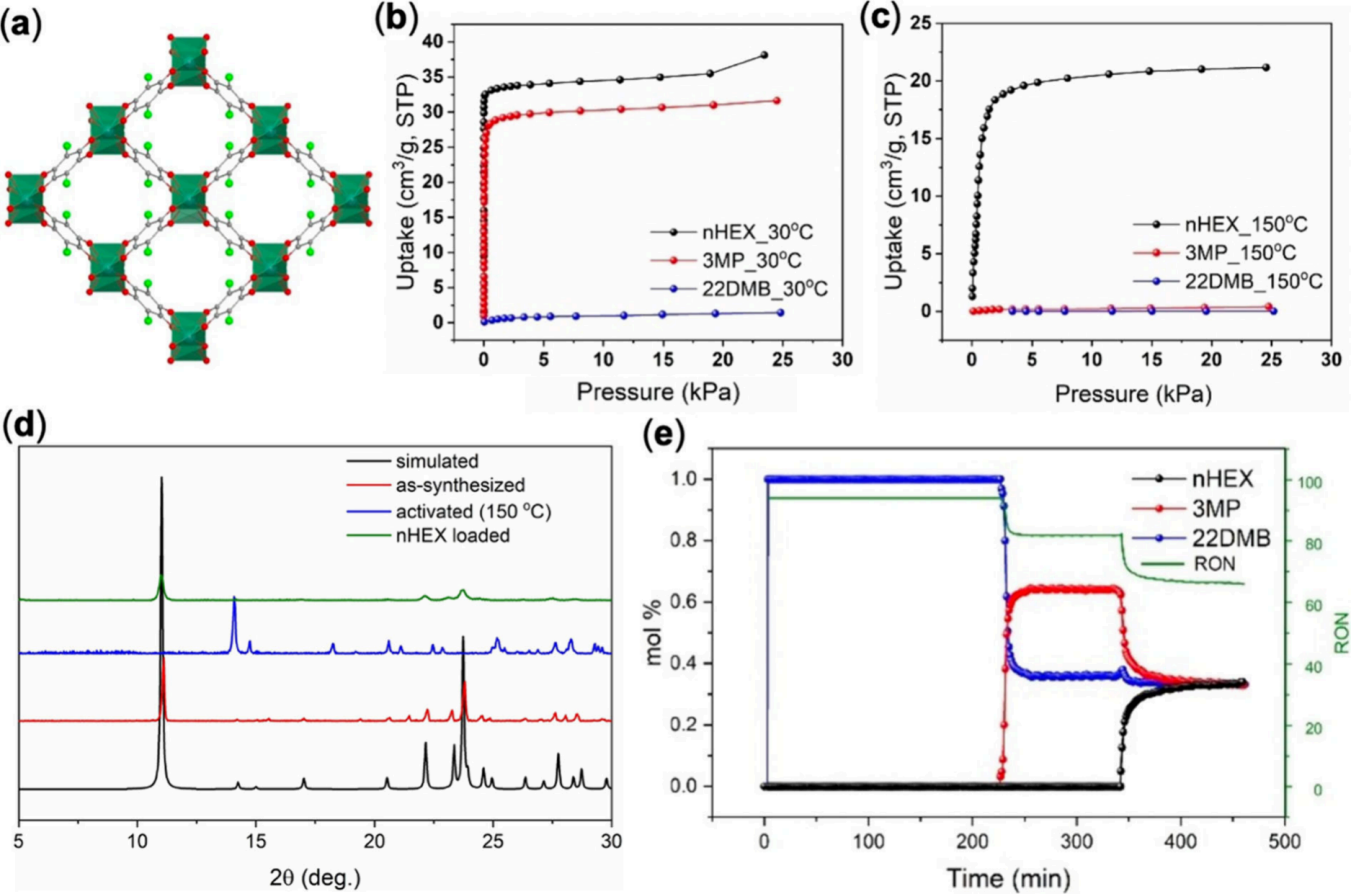
(a) Crystal structure of Ca-CHL. (b-c) Adsorption isotherms of hexane isomers on Ca-CHL at 30 and 150 °C. (d) PXRD patterns of Ca-CHL collected under different conditions. (e) Breakthrough curves of ternary mixture (nHEX/3MP/22DMB) using two columns for Ca-CHL at 150 and 30 °C. Reproduced with permission from ref 3. Copyright 2022, Wiley-VCH.

## 3 Separation of C8 Alkylaromatic Isomers

The separation of C8 alkylaromatics, including xylene isomers and ethylbenzene, is a key process with substantial economic implications for the chemical industry.^2,32^ These molecular species, predominantly obtained via catalytic reforming of crude oil or naphtha, serve as feedstocks for the manufacture of a wide range of chemicals. However, their similar properties often lead to the formation of azeotropic mixtures, making conventional distillation methods inefficient for their separation. Among the xylene isomers, p-Xylene is the most valuable, used as a crucial feedstock for producing terephthalic acid, a key precursor in the fabrication of poly­(ethylene terephthalate) and polyester. o-Xylene, essential for the production of phthalic anhydride, can be separated by distillation due to its slightly higher boiling point, although this process is energy-intensive and requires many stages. m-Xylene, integral to the production of isophthalic acid and its isomerization into other C8 compounds, presents even more challenges in separation. Adsorptive separation employing a simulated moving bed process is a preferred alternative for industrial-scale separation but suffers from low selectivity and slow adsorption kinetics.

The ability of FMOFs to discriminate among C8 aromatic isomers was discussed in several early reports.^36,37^ MIL-53, for example, is one of the most studied FMOFs exhibiting a marked preference for oX, with a selectivity following the order oX > mX > pX at low concentrations.^36,38^ However, the temperature dependence was not discussed in these studies. Given the subtle differences in molecular size, shape, and geometry among xylene isomers, FMOFs with highly dynamic structures are particularly attractive as they are sensitive to framework-guest interactions and temperature, which can effectively facilitate molecular-sieving based separations with the highest selectivity.

One prime example of our recent work is the investigation of the temperature-dependent adsorption behavior of a highly flexible 1D-MOF Mn-DHBQ (DHBQ = 2,5-dihydroxy-1,4-benzoquinone). The overall structure is a 3D hydrogen-bonded network (Figure 3a) made of 1D-Mn­(DHBQ)­(H_2_O)_2_ chains interconnected by a single type of H-bond (∼1.75 Å).^39^ The as-synthesized compound is nonporous but becomes porous upon activation to remove terminal water molecules, with an estimated BET surface area of ∼429 m^2^/g and a pore size of ∼5.6 Å. The activated sample contains high density of open metal sites (OMS) and abundant π-electrons.^2^ Selective adsorption of xylene isomers was observed at different temperatures (Figure 3b) associated with structure swelling (Figure 3e). Specifically, pX can expand and access the interchain spaces at all four temperatures due to its strongest interactions with the framework, whereas mX and oX cannot penetrate into the interchain spaces at elevated temperatures due to their weaker interactions. Only when the temperature decreases below a certain threshold can mX and oX be adsorbed. Based on their different adsorption behaviors, a complete separation of the three isomers was achieved via temperature-dependent molecular-sieving mechanism. Competitive vapor and liquid adsorption experiments were carried out on various binary and ternary xylene mixtures, and adsorption selectivities were obtained by proton nuclear magnetic resonance (^1^H NMR) (Figure 3c) and gas chromatography analysis. The results confirmed the capability of Mn-DHBQ for the highly selective recognition of specific xylenes at different temperatures. A sequential separation of all three isomers was achieved using a two-column breakthrough process at 90 and 30 °C (Figure 3d). Simulations reveal comparable π-π stacking interactions among three xylene isomers, with pX forming additional hydrogen bonds and stronger C···Mn dipole–dipole interactions compared to mX and oX, driving its preferential adsorption and high selectivity. Further investigation demonstrated its high performance in separating pX/oX/mX/EB (EB = ethylbenzene) quaternary liquid mixtures with record-high selectivities for pX at high temperatures. Moreover, the scaled-up synthesis of ∼0.23 kg Mn-DHBQ was achieved in a simple one-pot reaction at room temperature using water as the only solvent (Figure 3f). Coupled with its high adsorption capacity, excellent selectivity, and fast kinetics, Mn-DHBQ demonstrates substantial potential for real-world applications.

**Figure 3:**
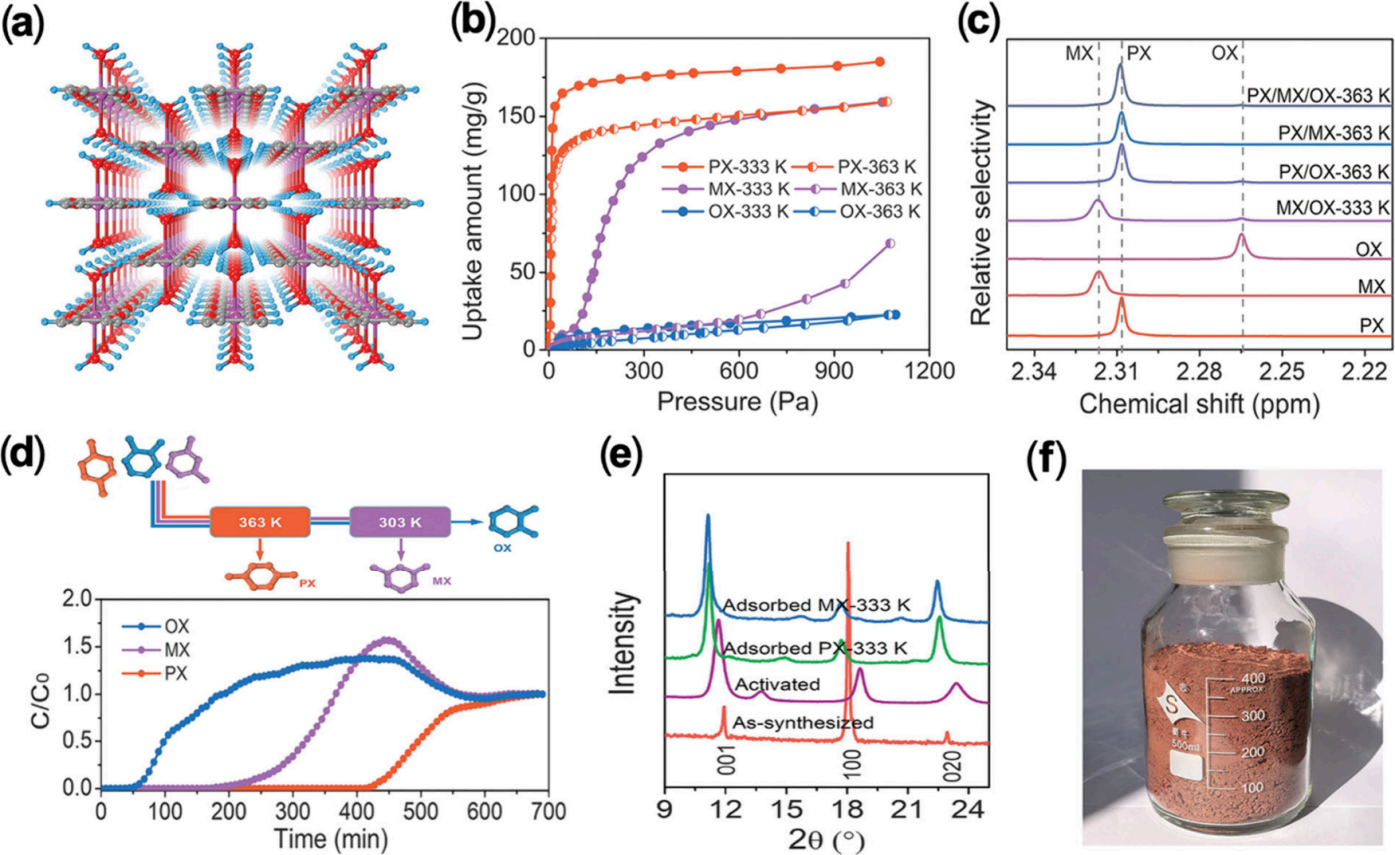
(a) Crystal structure of Mn­(DHBQ)­(H_2_O)_2_. (b) Vapor adsorption isotherms of xylene isomers on Mn­(DHBQ) at 60 and 90 °C. (c) Magnified ^1^H NMR spectrum recorded for the competitive vapor adsorption measurement of the equimolar binary or ternary vapor of xylene isomers on Mn-DHBQ at 60 and 90 °C. (d) Breakthrough curves for ternary pX/mX/oX using two columns on Mn­(DHBQ) at 90 and 30 °C. (e) PXRD patterns of various forms of Mn-DHBQ. (f) Scaled-up synthesis of the Mn-DHBQ product. Reproduced with permission from ref 2. Copyright 2022, American Association for the Advancement of Science.

It is interesting to note the inverse size-selective adsorption behavior of Mn-DHBQ toward some LHCs, where larger (aromatic) molecules are favored over smaller (nonaromatic) ones. For example, it can adsorb a high amount of both p-xylene and m-xylene (KD: 6.7 and 7.1 Å) while totally excluding smaller dibranched hexane isomers and cyclohexane (KD: 5.6 and 6.0 Å) at the same temperature (e.g., 60 °C). This unique reverse adsorption behavior observed in FMOFs arises from adsorbate-induced structural transformations, which is not possible for RMOFs, as their pore sizes remain constants (unchanged) upon adsorption, and therefore, separation based on sieving depends solely on the molecular dimensions of the adsorbates, where smaller molecules can enter the pore but larger molecules are excluded.

In a more recent study, we developed a 3D-FMOF (Figure 4a) Ca-CHL that achieved the highest selectivities for both pX/mX and pX/oX mixtures at 120 °C compared to all other MOFs reported to date (Figure 4f).^32^ It selectively adsorbs xylene isomers at different temperatures and at different rates. At 30 °C, all three isomers can be adsorbed (Figure 4b) but with significantly faster kinetics for pX. At 120 °C, only pX can be adsorbed in high capacity while both mX and oX are fully excluded (Figure 4c). This behavior allows efficient separation of pX from its isomers by different mechanisms at different temperatures: kinetic-driven processes at 30 °C and molecular sieving at 120 °C (Figure 4d,e). Framework flexibility, including linker tilting and volume expansion, plays a key role for its selective adsorption. Notably, Ca-CHL also exhibits inverse size-selective behavior similar to Mn-DHBQ: At 30 °C, larger xylene isomers (KD: > 6.5 Å) are fully adsorbed, while smaller dibranched hexane isomers (KD: < 6.2 Å) are excluded.

**Figure 4:**
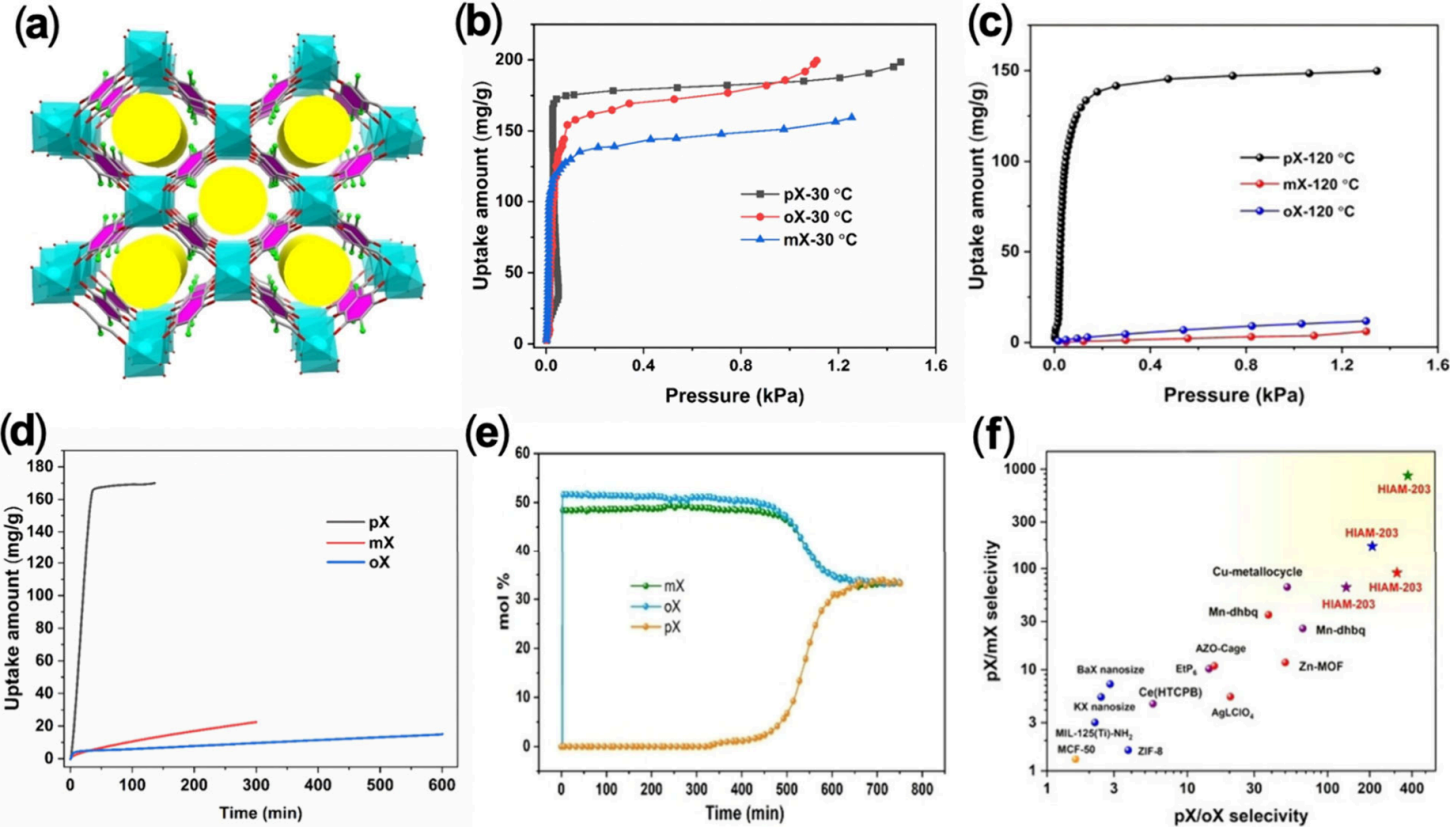
(a) Crystal structure of Ca-CHL. (b-c) Adsorption isotherms of xylene isomers for Ca-CHL at 30 and 120 °C. (d) Adsorption kinetics of xylene isomers at 30 °C. (e) Breakthrough curves for Ca-CHL at 120 °C for a ternary mixture of pX/mX/oX. (f) pX/oX and pX/mX selectivities of Ca-CHL and representative adsorbents at 120 °C. The values for Ca-CHL were obtained by four different methods. Reproduced with permission from ref 32. Copyright 2024, Wiley-VCH.

## 4 Separation of C6 Cyclic Hydrocarbons

The hydrogenation of benzene to yield cyclohexene or cyclohexane is a vital industrial process, as both are indispensable feedstocks for synthesizing cyclohexanol and cyclohexanone, which are used for generating adipic acid and caprolactam in manufacturing nylon.^22^ The final products from the catalytic hydrogenation of benzene contain both cyclohexene and cyclohexane, and their separation by simple distillation is inefficient due to the formation of azeotropic mixtures, resulting from their very similar boiling points and molecular geometries.^4^ Currently, large-scale separation relies on specialized distillation techniques, including multicolumn extraction and azeotropic distillation using solvents like dimethylacetamide and 1-methyl-2-pyrrolidone. However, these solvents suffer from low selectivity and limited solubility, therefore requiring multiple extraction cycles and resulting in substantial energy consumption and high equipment costs.

In 2007, Kitagawa et al. reported the first study on the adsorptive separation of benzene and cyclohexane by a FMOF, Zn-TCNQ-bpy, demonstrating the crucial role of ligand electronic environments in modulating adsorption affinity.^40^ A later study by Chen and co-workers used Cu­(etz) for the adsorptive separation of benzene and cyclohexane.^41^ However, these earlier studies were performed at constant temperatures, and as such, the temperature influence on the adsorption properties was not investigated. It was not until very recently when we began to explore temperature effect on the adsorption and separation of C6 cyclic HCs using FMOFs.^4,33^ We demonstrated that the 1D-FMOF, Mn-DHBQ (Figure 5a-5b), also acts as an very efficient adsorbent for the separation of binary and ternary mixtures of benzene, cyclohexene and cyclohexane via temperature-dependent molecular sieving processes.^4^ Our PXRD analysis confirmed that it undergoes fully reversible structural transformations between the as-synthesized, activated (water-removed), and hydrocarbon-loaded forms with a structural swelling effect. Its adsorption shows a sensitive dependence on temperature and adsorbate (Figure 5c-5d): Benzene is adsorbed at all three temperatures reaching equilibrium at low pressures, with a saturated uptake amount between 2.4 and 2.9 mmol/g, indicating its strong interaction and effective accommodation in the channels. Cyclohexane can only be partially adsorbed at 30 °C and totally excluded at both 60 and 90 °C, while the uptake of cyclohexene varies with temperature, reaching full adsorption at 30 °C (with essentially the same amount as benzene), ∼2.0 mmol/g at 60 °C but dropping to ∼0.1 mmol/g at 90 °C.

**Figure 5:**
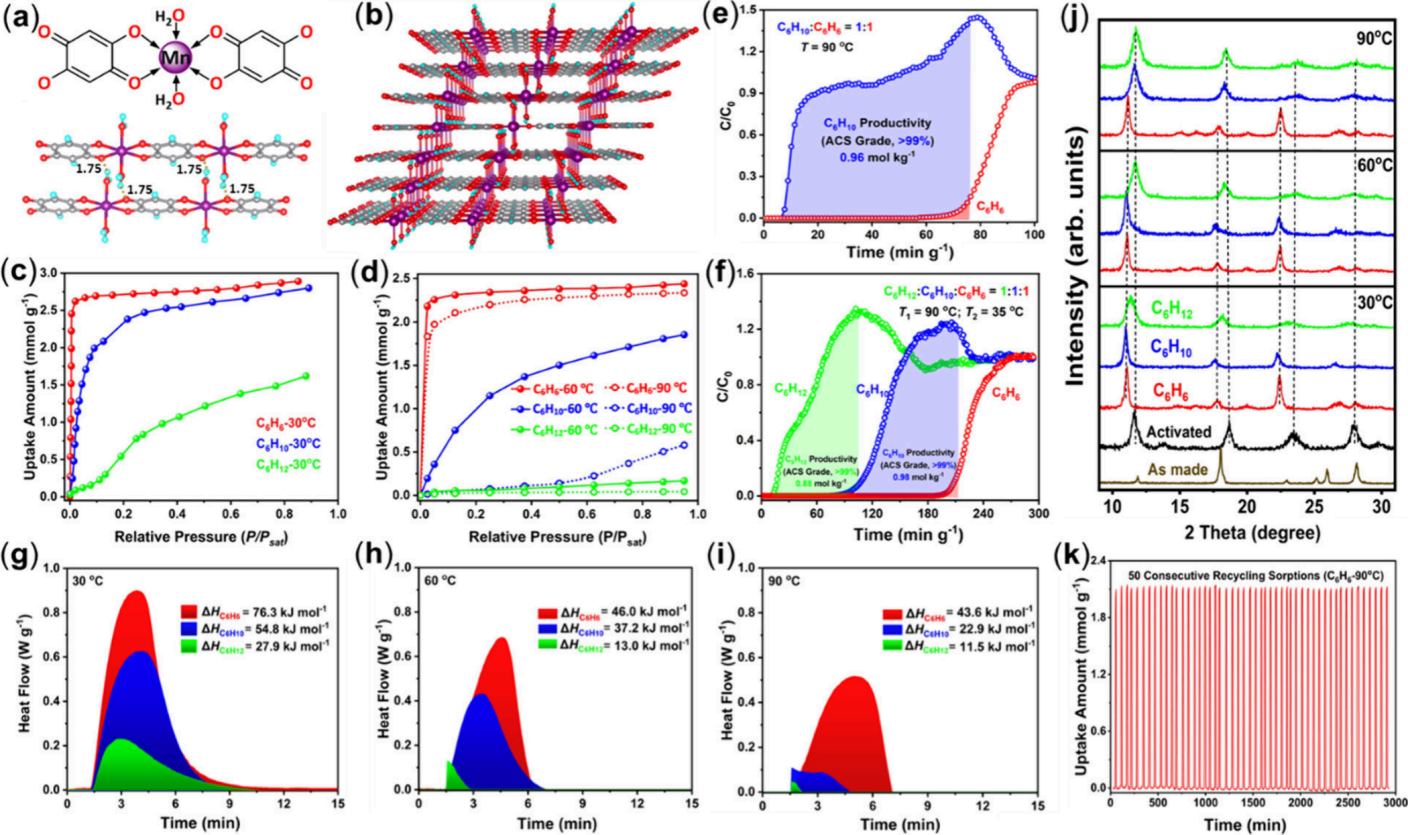
(a) Coordination environment of Mn metal and interchain hydrogen bonds (1.75 Å) between adjacent 1D chains. (b) Crystal structure of as-made Mn­(DHBQ)­(H_2_O)_2_. (c-d) Vapor adsorption isotherms of C6 cyclic hydrocarbons on Mn­(DHBQ) at 30, 60, and 90 °C. (e) Breakthrough curves for binary benzene/cyclohexene mixture using a single column setup at 90 °C, and (f) ternary benzene/cyclohexene/cyclohexane mixture using a two-column setup at 90 and 35 °C. The purity and productivity of the collected products are indicated in the figure. Heat flow and Δ*H* (<0) values obtained for three C6 cyclic hydrocarbons from independent TG-DSC measurements performed at (g) 30 °C, (h) 60 °C, and (i) 90 °C. (j) PXRD patterns of hydrocarbon-adsorbed Mn­(DHBQ) at various temperatures along with those of as-synthesized and activated samples. (k) Vapor phase recyclability test results of benzene on Mn­(DHBQ) for 50 consecutive adsorption–desorption cycles at 90 °C. Reproduced with permission from ref 4. Copyright 2024, Springer Nature.

The binding energies calculated by density functional theory are ∼54.0, 52.1, and 44.4 kJ/mol for benzene, cyclohexene, and cyclohexane, respectively. Clearly the extent of sorbate–sorbent interactions follows the order: C_6_H_6_ > C_6_H_10_ > C_6_H_12_, but the difference is relatively small. For a RMOF with a comparable pore size, this would typically lead to a thermodynamically driven process where all three species can be adsorbed with a somewhat different uptake amount. On the other hand, the experimentally measured heat changes or enthalpies (Δ*H*) associated with the structure swelling and adsorption processes are distinctly different for the three molecules and are strongly temperature dependent (Figure 5g-5i). It is interesting to note that certain energy is required to overcome the energy barrier and to open up the interchain space, allowing molecules to enter and to be adsorbed. With high Δ*H* values, benzene is able to expand the interchain space and enter the pore at all three temperatures. However, for cyclohexene at 90 °C and cyclohexane at 60/90 °C, the Δ*H* values are simply too small to provide sufficient energy to open up the pore space, so they are blocked out completely. A detailed PXRD analysis clearly reveals the correlation between the activated and guest-loaded structures and the extent of structure swelling as a function of the temperature (Figure 5j). The selective adsorption behavior of this FMOF enables a one-step full separation of benzene, cyclohexene, and cyclohexane via temperature-programmed molecular sieving process. Both cyclohexane and cyclohexene reaching the ACS grade were produced from both binary and ternary mixtures at a scale ranging from 0.77 to 1.0 mol/kg in a single cycle using either single- or two-column breakthrough experiments (Figure 5e-5f). Both the uptake amount of benzene and the crystal structure remained the same after 50 consecutive adsorption–desorption cycles at 90 °C (Figure 5k). With its simple one-pot and easily scalable synthesis, low-cost ligand, high stability, and excellent recyclability, Mn-DHBQ stands out as a promising candidate adsorbent for industrial separation of C6 cyclic hydrocarbons.

Our latest development is on a flexible MOF having a 2D layered structure. 2D-[Zn­(BDC)­(H_2_O)]·DMF (Zn-BDC, BDC = 1,4-benzenedicarboxylic acid) exhibits very similar temperature-dependent behavior toward adsorption of C6 cyclic hydrocarbons as Mn-DHBQ and acts as another efficient adsorbent for their separations via molecular-sieving. The structure of Zn-BDC consists of a Zn_2_(COO)_4_ paddle-wheel building unit and a simple dicarboxylate (BDC) linker, forming a square-like 2D network (Figure 6a). The 2D networks are interconnected by hydrogen bonds formed between terminal water molecules and carboxylate oxygens of DMF molecules.^42^ Upon activation, the compound loses both H_2_O and DMF molecules, undergoing a structure transformation, presumably forming stacked 2D layers of Zn­(BDC) (Figure 6b,c). The activated compound enables the selective adsorption of specific C6 cyclic hydrocarbons at different temperatures. As in the case of Mn-DHBQ, the as-made sample is nonporous but becomes porous upon activation with an estimated BET surface area of 308 m^2^/g and pore size of 5.4 Å. At 60 °C, only benzene is absorbed, while at 25 and 45 °C, cyclohexene can also be adsorbed. In contrast, the adsorption of cyclohexane is negligible at all three temperatures (Figure 6d). Differential scanning calorimetry experiments on Zn­(BDC) provide a quantitative measure of the heat involved in the structure expansion and adsorption processes of the three C6 cyclic hydrocarbons at different temperatures. The temperature-dependent adsorption behavior of Zn­(BDC), driven by structure expansion, enables efficient sieving-based separation of ternary mixtures of C6 cyclic hydrocarbons (Figure 6e). Notably, it reaches the second highest uptake ratio for C_6_H_10_/C_6_H_12_ and the third highest ratio for C_6_H_6_/C_6_H_12_, among all MOFs reported for separation of these species (Figure 6f). Again, it is interesting to note the inverse size-selective adsorption behavior of Zn­(BDC). At 25 °C, it takes up large amounts of xylene isomers (KD: 6.7–7.4 Å) but totally excludes mono- and dibranched *n*-hexane isomers (KD: 5.0–6.2 Å) and cyclohexane (KD: 6.0 Å). This study is the first to demonstrate the adsorptive separation of three C6 cyclic hydrocarbons using a flexible 2D layered MOF sorbent.

**Figure 6:**
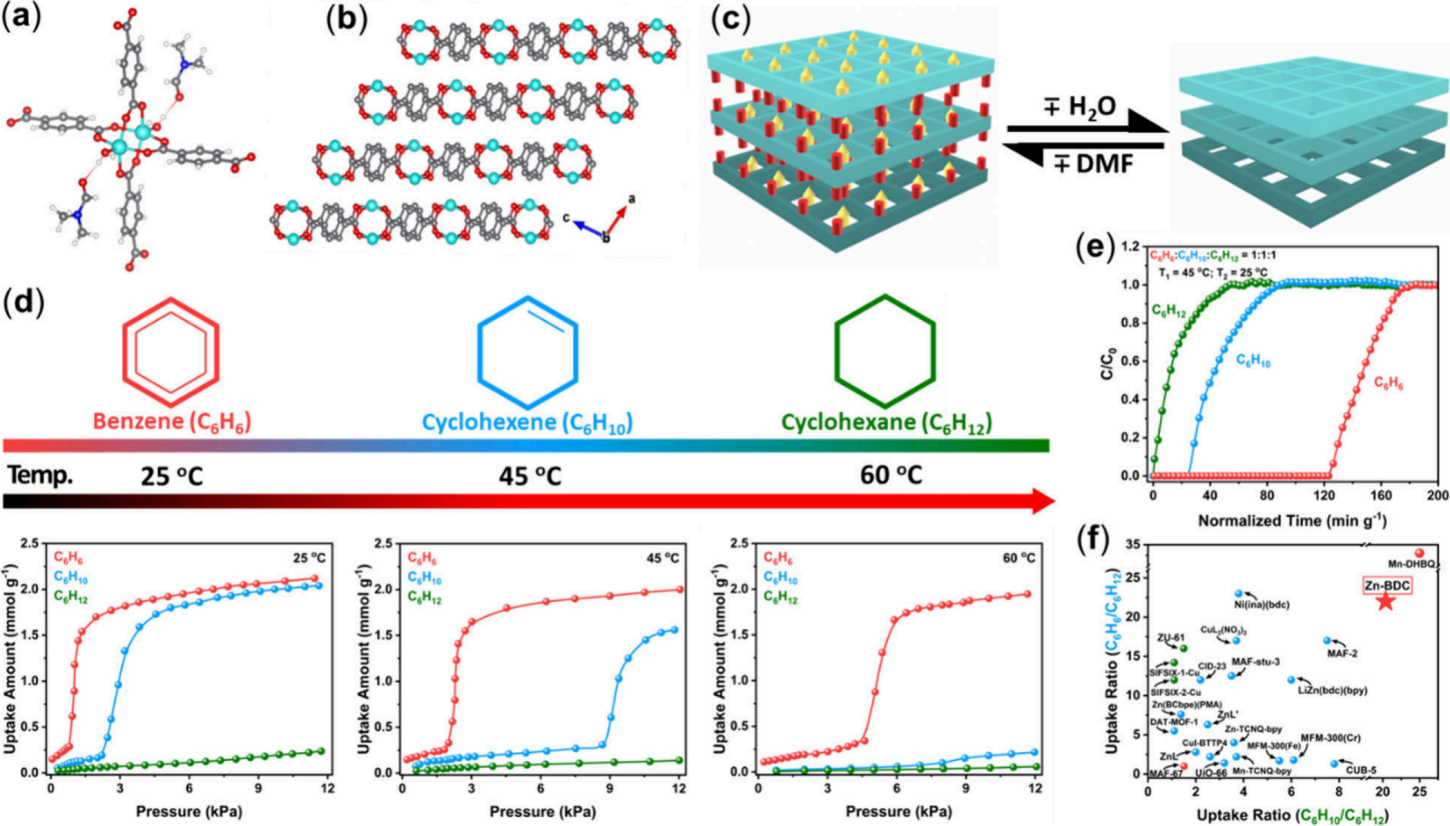
(a) The Zn_2_(COO)_4_ paddle-wheel building unit in the Zn-BDC structure. (b) The 2D stacked layers viewed along the *b*-axis. (c) Schematic representation of the reversible structure changes between the as-made and activated 2D structure. (d) Temperature-dependent vapor adsorption isotherms of C6 cyclic hydrocarbons in Zn­(BDC). (e) Breakthrough curves for temperature-programmed separation of an equimolar ternary mixture of C6 cyclic hydrocarbons using a two-column setup at 45 and 25 °C on Zn­(BDC). (f) Comparison of the separation performance of Zn­(BDC) with the best-performing MOFs. Reproduced with permission from ref 33. Copyright 2025, American Chemical Society.

## 5 Conclusion and Perspective

FMOFs hold significant promise for the energy-efficient separation and purification of hydrocarbons with very similar properties. Unlike RMOFs, all FMOFs we have investigated exhibit transformable and reversible crystal structures across different sample forms: as-synthesized, activated, and hydrocarbon-loaded. Leveraging their structural flexibility and unique adsorption properties, they outperform other sorbent materials, including RMOFs, by simultaneously achieving high adsorption capacity, exceptional selectivity, and fast diffusion rate, overcoming the trade-offs typically encountered by sorbents with rigid structures such as RMOFs.

In this *Account*, we summarize our recent advancements in using FMOFs for the highly selective separation of three key groups of LHCs: C6 alkane isomers, C8 alkylaromatic isomers, and C6 cyclic hydrocarbons—where conventional separation technologies struggle with low performance. Our discussions focus on their interesting temperature- and adsorbate-dependent adsorption behaviors and how these properties enable molecular sieving with unparalleled selectivity. Special emphasis is placed on the correlation between their highly dynamic, fully reversible structures and their adsorption performance and how their flexible frameworks enable fast mass transport without compromising selectivity, effectively minimizing the trade-offs among three key parameters: capacity, diffusion, and selectivity. The following aspects have been considered in our selection of FMOF structures: 1) dimensionality. In general, the structural flexibility of a framework decreases as the dimensionality increases (1D > 2D > 3D). FMOFs with 1D chain or 2D layered structures are usually more flexible compared to 3D frameworks and have a higher degree of expandability, facilitating structure expansion/swelling with lower energy requirement; 2) pore size. FMOFs with pore sizes (of the activated forms) slightly smaller than the kinetic diameters of the target guest molecules are chosen. This mismatch can effectively trigger and control framework expansion via guest-induced flexibility and sorbate-dependent adsorption at different temperatures, facilitating molecular-sieving based separation of LHCs with similar dimensions. For example, the pore sizes of activated 1D-Mn­(DHBQ) and 2D-Zn­(BDC) are ∼5.5 and ∼5.3 Å, respectively. Both are smaller than the kinetic diameters of the targeted LHCs molecules (e.g., benzene, cyclohexene, and cyclohexane at ∼5.85–6.0 Å; xylene isomers at ∼6.7–7.4 Å), suggesting significant interchain or interlayer swelling/expansion.

Despite notable progress, significant challenges and unanswered questions remain before the observed phenomena can be fully understood. A major hurdle is the lack of precise structure information on FMOFs in activated and hydrocarbon-loaded states, as few crystal structures have been determined due to reduced crystallinity and disorder. Another key issue is that structural transformation and adsorption are inherently entangled, and the energies involved in the two processes cannot be experimentally separated. The complexity of such systems makes it difficult to analyze and understand the host–guest and guest–guest interactions within their dynamic frameworks. Developing accurate diffusion and mass transfer models, as well as state-of-the-art *in situ* analytical tools, are essential to reveal structural details in all crystal forms, advance mechanistic understanding, and optimize FMOF performance. In particular, in situ synchrotron PXRD and neutron scattering techniques have emerged as powerful tools, offering high-resolution structural insights under operating conditions and enabling real-time monitoring of phase transitions, framework expansion and contraction, and guest adsorption pathways. Additionally, current theoretical calculations fall short in simulating the flexible framework and dynamic adsorption behavior of FMOFs when crystal structures are unavailable. Advancing computational methods for accurate modeling and prediction is crucial^43^ in providing guidelines for designing high-performance flexible structures and predicting adsorption behavior of FMOFs, including the influence of dimensionality, metal centers, and ligands. Moreover, while substantial progress has been made in vapor-phase separations, liquid-phase LHCs separation remains largely underexplored. Expanding studies to liquid-phase conditions is critical for evaluating FMOFs’ industrial applicability. Pilot experiments under high-temperature and high-pressure conditions that mimic industrial settings will be instrumental. Equally importantly, developing ecofriendly, cost-effective, and easily scalable synthesis protocols is crucial for the sustainable commercialization of FMOFs. This includes using inexpensive, nontoxic reagents and precursors and adopting solvent-free, aqueous-phase, and other green synthesis methods under mild conditions to enable industrial-scale production.

Finally, it is important to recognize that certain limitations may exist in the separation of LHCs by FMOFs, such as operating conditions (e.g., pressure and temperature), which must align with their adsorption behavior. Thus, efforts should focus on optimizing the structural dynamics of FMOFs to achieve the desired separation performance under industrially relevant conditions. Stronger collaboration between academia and industry will be crucial for deepening our understanding of structure–property relationships and for accelerating innovation. Addressing these challenges systematically unlocks the full potential of FMOFs, positioning them as competitive adsorbents capable of revolutionizing traditional separation technologies, including LHCs separations discussed in this work.
